# AGROBEST: an efficient *Agrobacterium*-mediated transient expression method for versatile gene function analyses in *Arabidopsis* seedlings

**DOI:** 10.1186/1746-4811-10-19

**Published:** 2014-06-18

**Authors:** Hung-Yi Wu, Kun-Hsiang Liu, Yi-Chieh Wang, Jing-Fen Wu, Wan-Ling Chiu, Chao-Ying Chen, Shu-Hsing Wu, Jen Sheen, Erh-Min Lai

**Affiliations:** 1 Institute of Plant and Microbial Biology, Academia Sinica, Taipei 11529, Taiwan; 2 Department of Plant Pathology and Microbiology, National Taiwan University, Taipei 10617, Taiwan; 3 Department of Molecular Biology and Center for Computational and Integrative Biology, Massachusetts General Hospital, Boston MA 02114, USA; 4 Department of Genetics, Harvard Medical School, Boston, MA 02114, USA; 5 Center for the Study of Biological Complexity, Virginia Commonwealth University, Richmond, VA 23284, USA

**Keywords:** *Agrobacterium*, *Arabidopsis*, Transient transformation, Gene expression, Innate immunity, Gain-of-function

## Abstract

**Background:**

Transient gene expression via *Agrobacterium*-mediated DNA transfer offers a simple and fast method to analyze transgene functions. Although *Arabidopsis* is the most-studied model plant with powerful genetic and genomic resources, achieving highly efficient and consistent transient expression for gene function analysis in *Arabidopsis* remains challenging.

**Results:**

We developed a highly efficient and robust *Agrobacterium*-mediated transient expression system, named AGROBEST (*Agrobacterium*-mediated enhanced seedling transformation), which achieves versatile analysis of diverse gene functions in intact *Arabidopsis* seedlings. Using β-glucuronidase (GUS) as a reporter for *Agrobacterium-*mediated transformation assay, we show that the use of a specific disarmed *Agrobacterium* strain with *vir* gene pre-induction resulted in homogenous GUS staining in cotyledons of young *Arabidopsis* seedlings. Optimization with AB salts in plant culture medium buffered with acidic pH 5.5 during *Agrobacterium* infection greatly enhanced the transient expression levels, which were significantly higher than with two existing methods. Importantly, the optimized method conferred 100% infected seedlings with highly increased transient expression in shoots and also transformation events in roots of ~70% infected seedlings in both the immune receptor mutant *efr-1* and wild-type Col-0 seedlings. Finally, we demonstrated the versatile applicability of the method for examining transcription factor action and circadian reporter-gene regulation as well as protein subcellular localization and protein–protein interactions in physiological contexts.

**Conclusions:**

AGROBEST is a simple, fast, reliable, and robust transient expression system enabling high transient expression and transformation efficiency in *Arabidopsis* seedlings. Demonstration of the proof-of-concept experiments elevates the transient expression technology to the level of functional studies in *Arabidopsis* seedlings in addition to previous applications in fluorescent protein localization and protein–protein interaction studies. In addition, AGROBEST offers a new way to dissect the molecular mechanisms involved in *Agrobacterium*-mediated DNA transfer.

## Background

*Agrobacterium*-mediated DNA transfer is currently the most facile and versatile method to deliver gene constructs into the nucleus for gene function analysis in diverse plant species [1-3]. Although stable integration of physiologically active and regulated transgenes is the ultimate goal, transient gene expression via *Agrobacterium*-mediated DNA transfer in different plant tissues offers a simple and fast method to analyze transgene functions, which is amenable for high-throughput screens. The transient expression assay is also ideal for systematic dissection of the exquisite and complex processes of *Agrobacterium*–plant interactions and DNA transfer events [4-7].

*Agrobacterium tumefaciens* is a soil phytopathogen that naturally infects plant wound sites and causes crown gall disease via delivery of transferred (T)-DNA from bacterial cells into host plant cells through a bacterial type IV secretion system (T4SS) [8]. Although *Agrobacterium* is considered a wound-associated pathogen, it can transfer DNA into diverse host cells or tissues under unwounded conditions [9-13]. Interestingly, most of the *Arabidopsis* mutants that are resistant to *Agrobacterium* transformation identified by root explant assays remain highly transformable by floral dip transformation [14]. The mechanisms and plant factors involved in *Agrobacterium-*mediated transformation may differ between wounded and unwounded cells or different tissues. However, the mechanisms underlying *Agrobacterium* infection in unwounded cells/tissues have not been explored.

In plant biology research, *Arabidopsis* mesophyll-protoplast transfection [15,16] and *Agrobacterium-*mediated leaf infiltration in *Nicotiana benthamiana*[17] are the well-established and commonly used platforms for transient gene expression analysis. The *Arabidopsis* mesophyll-protoplast transient expression system allows for versatile and high-throughput analyses of diverse gene functions and signal transduction pathways; advanced skills with training and practice are essential for successful use of this powerful tool for gene function studies [16,18,19]. *Agrobacterium-*mediated transient expression methods by leaf infiltration have been developed for a wide range of plants including *Nicotiana*, lettuce, tomato, and *Arabidopsis*[20-23]. However, the use of 4- to 5-week-old adult plants with manual infiltration has limited application in high-throughput analyses. Furthermore, although *Arabidopsis* is the most-studied model plant with superbly annotated genome sequences and powerful genetic and genomic resources mostly available for the Columbia (Col) accession, achieving highly efficient and consistent transient expression in Col by adult leaf infiltration is challenging [22,24].

The use of young seedlings for *Agrobacterium*-mediated transient expression assays will greatly simplify and amplify the power of the method. Indeed, *Agrobacterium*-mediated transient expression in *Arabidopsis* seedlings has been recently developed for fast and robust analysis of protein subcellular localization and protein–protein interactions [25-27]. The system’s requirement for high-density *Agrobacterium* cells and vacuum infiltration [27] or chemical treatment (e.g., the addition of surfactant Silwet L-77) [26] to achieve high cellular transformation efficiency could induce innate immunity and stress responses in plants, which globally alters cellular, physiological, and signaling processes and severely retards growth [28,29]. Thus, developing a system that circumvents a plant defense barrier may be a key to enhance transient expression efficiency in *Arabidopsis* seedlings. Furthermore, such a fast, robust, and highly efficient transient expression system could support gain-of-function studies of diverse genes and signaling pathways *in planta*.

Pattern-triggered immunity (PTI) induced by a microbe- or pathogen-associated molecular pattern (MAMP or PAMP) is the first line of active defense in both plants and animals against pathogens [28-30]. Previous studies have suggested that *Agrobacterium-*mediated transformation efficiency may be compromised when plants recognize *Agrobacterium* MAMPs by corresponding pattern-recognition receptors (PRRs) to trigger PTI and block *Agrobacterium* infection [22,24]. The elongation factor Tu (EF-Tu) receptor mutant *efr-1*, which cannot sense EF-Tu MAMP, showed increased *Agrobacterium-*mediated transient expression efficiency, as did transgenic *Arabidopsis* expressing a potent bacterial effector AvrPto to suppress PTI signaling with agroinfiltration of 4- to 5-week-old leaves [22,24]. However, whether these immune-compromised *Arabidopsis* plants are amenable to increase *Agrobacterium-*mediated transient expression efficiency in young seedlings has not been tested. Defining the condition for reliable and highly efficient transformation in healthy Col-0 seedlings will be extremely valuable but has never been achieved.

In this study, we systematically investigated various biological factors and growth variances to define a combination of key factors that contribute to the unprecedentedly high transient transformation and reporter gene expression efficiency in *Arabidopsis* seedlings. As a result of these investigations, we developed an optimized AGROBEST (*Agrobacterium*-mediated enhanced seedling transformation) method that enabled high transient transformation and expression efficiency in both *efr-1* mutant and Col-0 *Arabidopsis* seedlings. Importantly, we demonstrated the versatile applicability of AGROBEST in gain-of-function studies for the MYB75 transcription factor in specific target-gene activation and for *GIGANTEA* (*GI*) reporter gene expression regulated by the *Arabidopsis* circadian clock. The AGROBEST method is a fast, simple, reliable, and versatile tool for systematic gene function analysis and a new tool for dissecting the *Agrobacterium*-mediated DNA transfer processes.

## Results

### Cotyledons of young *Arabidopsis* EF-TU receptor mutant is highly susceptible to *Agrobacterium*-mediated transient transformation

**Figure 1:**
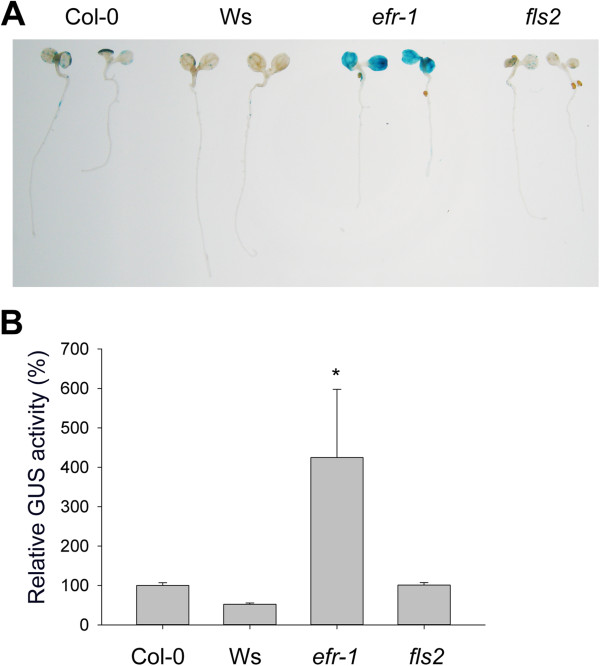
**Transient transformation assays in different *Arabidopsis *ecotype/genotypes.** Four-day-old *Arabidopsis* seedlings were infected with *Agrobacterium* strain C58C1(pTiB6S3ΔT)^H^ carrying pBISN1, which was pre-incubated in AB-MES (pH5.5) supplemented with 200 μM acetosyringone (AS) to induce *vir* gene expression. Seedlings were co-cultivated with pre-induced *A. tumefaciens* cells with final OD_600_ = 0.02 in the MS medium (1/2 MS, 0.5% sucrose (w/v), pH 5.5) containing 50 μM AS and determined for transient GUS expression levels by overnight GUS staining **(A)** and quantitative GUS activity **(B)** at 3 dpi. The GUS activity obtained from Col-0 seedlings was set to 100% and that of Ws, *efr-1*, and *fls2* is relative to that of Col-0. Data are mean ± SD GUS activity from two biological replicates. Similar results were obtained from at least two independent experiments. Values significantly different from that obtained with Col-0 are denoted (*P = 0.058 by Student’s *t* test).

Environmental and biological factors such as growth conditions, host plants, and *Agrobacterium* strains can affect the transformation efficiency. We first evaluated the transient expression efficiency of selected *Arabidopsis* ecotypes and mutants defective in pattern-recognition receptors (PRRs) with a disarmed *A. tumefaciens* strain C58C1(pTiB6S3ΔT-DNA) [31] containing a pCH32 helper plasmid [32] and abbreviated as C58C1(pTiB6S3ΔT)^H^. The T-DNA vector pBISN1 harboring the *gusA-intron*[12] was transformed into C58C1(pTiB6S3ΔT)^H^ to infect 4-d-old seedlings, and β-glucuronidase (GUS) activity was determined to monitor transient expression efficiency at 3 days post-infection (dpi). We consistently observed 100% of analyzed EF-Tu receptor mutant *efr-1* seedlings were successfully transformed, with strong and homogenous GUS staining in cotyledons, with 4-fold higher GUS activity in *efr-1* than wild-type Col-0 seedlings (Figure 1A and B, Additional file 1: Table S1). The flagellin receptor mutant, *fls2*, and the Ws ecotype that is highly susceptible to *Agrobacterium* transformation in the root explant [14] and a natural *fls2* variant [33] showed similar transient GUS expression efficiency as the Col-0, so the *fls2* mutant contributes little to enhancing *Agrobacterium*-mediated transient transformation (Figure 1A and B). Our seedling transient expression results confirm and further support that EFR but not FLS2 is an important factor limiting *Agrobacterium-*mediated transient expression efficiency previously observed by agroinfiltration of *Arabidopsis* adult leaves [24,34,35].

### Buffered medium at pH 5.5 with AB salts is critical for high transient expression efficiency

**Figure 2:**
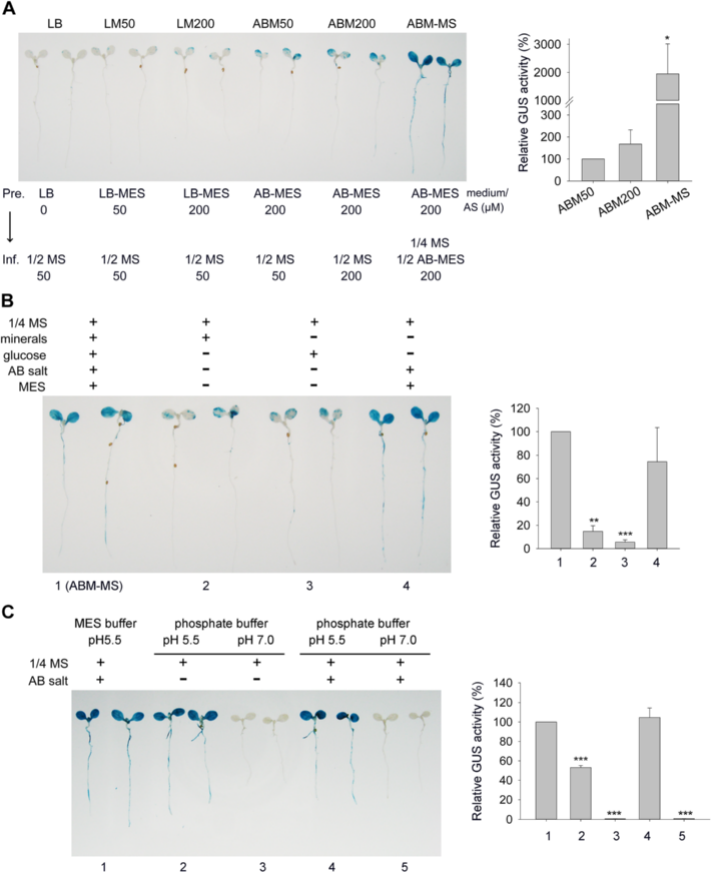
**Optimization of *Agrobacterium *pre-culture and infection media for efficient transient expression efficiency.** Four-day-old *Arabidopsis efr-1* seedlings infected with *Agrobacterium* C58C1(pTiB6S3ΔT)^H^ carrying pBISN1 were grown in various pre-culture and co-cultivation media to test their effects on transient GUS expression efficiency measured by GUS staining and quantitative GUS activity. **(A)** Various pre-culture and infection media in the absence or presence of *vir* gene inducer AS at the indicated concentration. **(B)** Effect of factors in AB-MES medium on increased transient expression efficiency. **(C)** Effect of AB salts, pH and buffering systems on transient GUS expression efficiency. Data for relative quantitative GUS activity are mean ± SD of 3 independent experiments. Values significantly different from that infected by ABM50 **(A)** or condition 1 **(B and C)** are denoted (*P < 0.05, **P < 0.01, ***P < 0.005 by Student’s *t* test).

To exploit this transient expression system for higher efficiency, we tested several factors including pre-induction and co-cultivation conditions. Pre-induction with acetosyringone (AS) in AB-MES medium (ABM50 and ABM200 methods) and continuous addition of AS to stimulate *vir* gene expression during the infection process are required for efficient transient GUS expression. Because AB-MES medium (pH 5.5) is the optimized medium for *vir* gene induction [36,37], we tested whether mixing AB-MES (pH 5.5) with an equal volume of commonly used plant culture MS medium (1/2 MS, 0.5% sucrose (w/v), pH 5.5), named ABM-MS (1/2 AB-MES, 1/4 MS, 0.25% sucrose (w/v), pH 5.5) in the presence of AS could produce high transient expression efficiency. Strikingly, GUS activity was strongly expressed in all seedlings and was 20-fold higher with co-cultivation in ABM-MS than in MS medium alone (Figure 2A, Additional file 1: Table S1). To avoid over-staining, the reaction time for histological GUS staining shown in Figure 2 was limited to 6 hr instead of overnight for the result in Figure 1A.

Key components in AB-MES are AB salt (17.2 mM K_2_HPO_4_, 8.3 mM NaH_2_PO_4_, 18.7 mM NH_4_Cl, 2 mM KCl), minerals (1.25 mM MgSO_4_, 100 μM CaCl_2_, 10 μM FeSO_4_), glucose (2% w/v), and buffering with MES (50 mM) to pH 5.5. We thus tested whether one of these components is responsible for the increased transient expression efficiency. The addition of AB salts with MES buffered at pH 5.5 in MS medium was sufficient to result in comparable levels of GUS expression as with ABM-MS (Figure 2B). Therefore, AB salts alone, pH 5.5 buffered by MES, or both, are critical for the increased transient expression efficiency. Strikingly, all MS media with the addition of AB salts buffered with MES or sodium phosphate at pH 5.5 showed comparable and strong GUS activity as that with ABM-MS (Figure 2C). However, omitting AB salts resulted in ~50% reduction in GUS activity, and no GUS activity was detected with MS medium buffered with sodium phosphate at pH 7.0 in the presence or absence of AB salts. Thus, buffered pH at 5.5 and the presence of AB salts in MS co-cultivation medium are the two key factors for this high transient expression efficiency. We named this optimized infection method AGROBEST (*Agrobacterium*-mediated enhanced seedling transformation).

### Disarmed *Agrobacterium* strain C58C1(pTiB6S3ΔT)^H^ enables highly efficient AGROBEST-mediated transient expression in Col-0 seedlings

**Figure 3:**
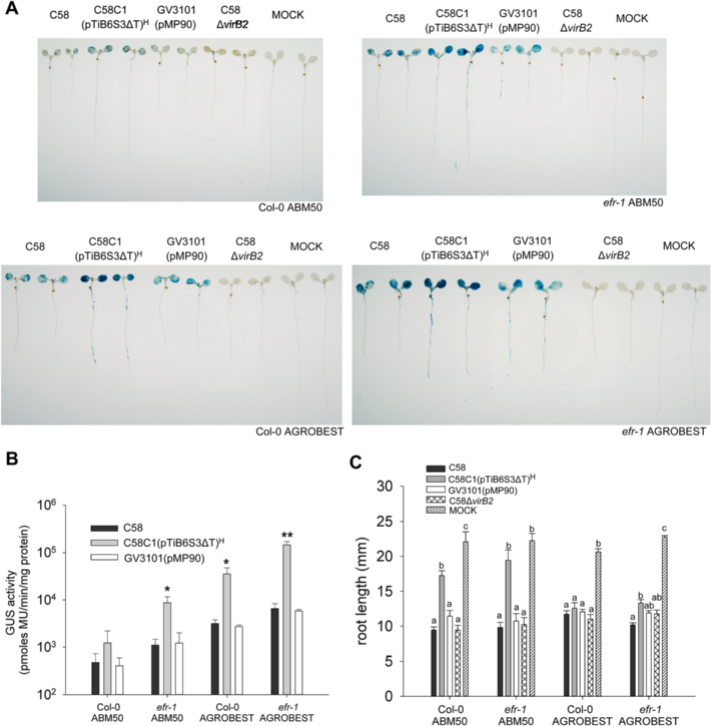
**Transient transformation of the *Arabidopsis *seedlings by various *Agrobacterium *strains.** Four-day-old *Arabidopsis* Col-0 and *efr-1* seedlings infected with different *Agrobacterium* stains carrying pBISN1 by ABM50 or ABM-MS (named as AGROBEST) were compared by GUS staining **(A)**, quantitative GUS activity **(B)**, and root length **(C)** at 3 days post-inoculation (dpi). Data for quantitative GUS activity are mean ± SD of at least 4 biological replicates from 2 independent experiments. Values significantly different from that infected with wild-type C58 are denoted (*P < 0.05, **P < 0.01 by Student’s *t* test). Data for root length measurement are mean ± SEM of 4-6 biological replicates from 2–4 independent experiments. Statistics was analyzed by ANOVA and means annotated with the same letter (a-c) are not significantly different; those with different letters are significantly different (*P* < 0.05). Seedlings grown in the same co-cultivation medium without *Agrobacterium* infection are indicated (MOCK).

Next, we tested whether the AGROBEST method optimized with *efr-1* seedlings could also improve *Agrobacterium*-mediated transient transformation in wild-type Col-0 seedlings. Because the use of C58C1(pTiB6S3ΔT)^H^ as compared with other disarmed or virulent *A. tumefaciens* strains produced higher transient expression levels with leaf agroinfiltration of various plants [23], we also tested whether C58C1(pTiB6S3ΔT)^H^ is a more superior strain in our system. We compared C58C1(pTiB6S3ΔT)^H^ with the wild-type virulent strain C58 or C58-derived disarmed strain GV3101(pMP90) [38] for their transient expression efficiency in *efr-1* and Col-0 seedlings using both sub-optimal ABM50 and optimized AGROBEST methods. Remarkably, Col-0 seedlings infected by all transfer-competent strains achieved significantly higher transient expression efficiency by AGROBEST than ABM50 (Figure 3A and B). Moreover, Col-0 seedlings infected by AGROBEST showed higher transient expression than *efr-1* seedling infected by ABM50 (Figure 3A and B). No GUS stains could be detected in control seedlings without infection (MOCK) or infected with Δ*virB2*, a strain lacking the key component of the type IV secretion system (T4SS) essential for T-DNA/effector translocation [8,39]. Therefore, the GUS activity detected was indeed from T-DNA gene expression inside the plant cells. Strikingly, 5- to 15-fold higher GUS activity was observed in *efr-1* or Col-0 seedlings infected with C58C1(pTiB6S3ΔT)^H^ than with C58 or GV3101(pMP90) (Figure 3A and B). The root length was significantly shorter for *Arabidopsis* seedlings infected with C58, Δ*virB2*, or GV3101(pMP90) at 3 dpi in all combinations or with C58C1(pTiB6S3ΔT)^H^ by the AGROBEST method as compared with uninfected seedlings (MOCK) (Figure 3C). Notably, seedlings infected with C58C1(pTiB6S3ΔT)^H^ showed no or little inhibition of root elongation in *efr-1* seedlings with the AMB50 method, which achieves fair although not the highest transient expression efficiency.

### AGROBEST achieves higher transient expression efficiency than existing methods in both *efr-1* and Col-0 seedlings

**Figure 4:**
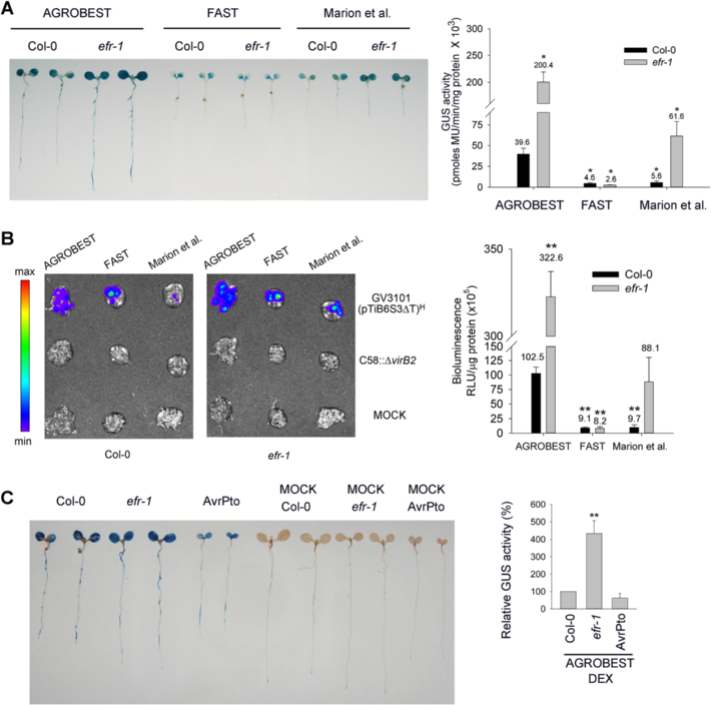
**AGROBEST enables high transient expression levels in Col-0.** Four-day-old *Arabidopsis* seedlings were infected with *Agrobacterium* strain C58C1(pTiB6S3ΔT)^H^ carrying pBISN1 **(A and C)** or 35S::*LUC2***(B)**, and transient expression activity was determined at 3 dpi. **(A)** Transient GUS expression efficiency of Col-0 and *efr-1* seedlings by AGROBEST, FAST and Marion et al. methods. Data for quantitative GUS activity are mean ± SD of 3 biological replicates. Values significantly different from those obtained with Col-0 by AGROBEST are denoted (*P < 0.05 by Student’s *t* test). **(B)** Transient luciferase expression efficiency of Col-0 and *efr-1* seedlings by AGROBEST, FAST and Marion et al. methods. Seedlings infected by C58Δ*virB2* carrying 35S::*LUC2* were used as a background control and those without *Agrobacterium* infection are indicated as MOCK. Luciferase activity of Col-0 obtained by AGROBEST was set to 100% and that of others is relative to activity of Col-0 by AGROBEST. Data are mean ± SD of 3 biological replicates. Values significantly different from those obtained with Col-0 by AGROBEST are denoted (**P < 0.01, by Student’s *t* test). **(C)** Transient GUS expression efficiency of Col-0, AvrPto transgenic line, and *efr-1* by AGROBEST. For dexamethasone (DEX) treatment, 3-d-old seedlings were treated with 10 μM DEX for 1 day and the following 3 days infected by the AGROBEST method. Quantitative GUS activity from DEX-induced Col-0 seedlings by AGROBEST was set to 100% and that of others is relative to activity of DEX-induced Col-0 seedlings with AGROBEST. Data are mean ± SD GUS activity from 4 repeats (2 biological repeats from each of 2 independent experiments). Values significantly different from that obtained with Col-0 are denoted (**P < 0.01 by Student’s *t* test). Seedlings grown in the same co-cultivation medium without *Agrobacterium* infection are indicated (MOCK).

We also compared AGROBEST with previously developed methods [26,27] for their transient expression efficiency in both Col-0 and *efr-1* seedlings. Remarkably, all Col-0 seedlings infected by the AGROBEST showed ~10-fold increased transient expression efficiency than with two existing methods, the FAST method [26] and the method by Marion et al. [27] with either GUS (Figure 4A) or luciferase (*LUC2*) (Figure 4B) used as reporters. Interestingly, both AGROBEST and the Marion et al. method achieved significantly higher transient expression activity in *efr-1* than in Col-0, *efr-1* seedlings remained poorly transformed by the FAST method (Figure 4A and B). As a result, AGROBEST conferred at least 40-fold and 3-fold higher transient expression efficiency in *efr-1* seedlings than with FAST and the Marion et al. methods, respectively (Figure 4A and B). However, we detected no increased transient expression activity in infected seedlings of the dexamethasone (DEX)-induced AvrPto transgenic line than in Col-0 seedlings with the AGROBEST method (Figure 4C), despite a significantly higher transient expression efficiency than Col-0 detected in adult leaves by agroinfiltration [22]. The AvrPto transgenic line germinated at the same rate and grew to a similar size as Col-0 and *efr-1*, but growth was arrested with the addition of DEX at 3 days old. This finding is consistent with previous studies showing that overexpression of AvrPto can also interfere with growth hormone signals and trigger cell death by interrupting the diverse functions of BAK1 and BKK1 in multiple receptor complexes, not restricted to PRRs [40].

### Impact of seedling age and infection time on transient expression efficiency of AGROBEST in *efr-1* seedlings

**Figure 5:**
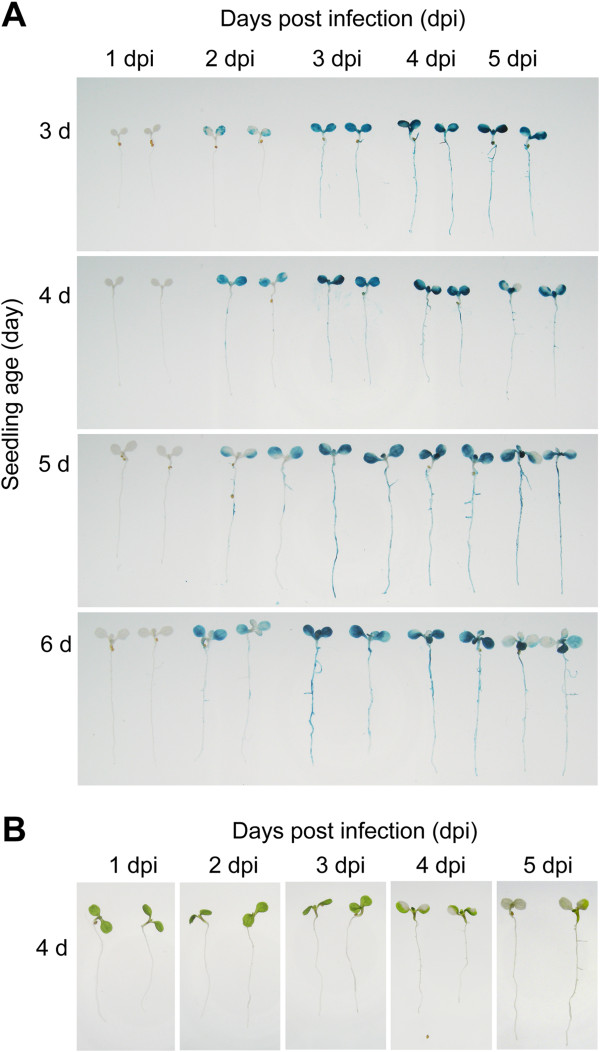
**Impact of seedling age and infection time on transient expression. (A and B)** Different ages of *Arabidopsis efr-1* seedlings were infected with C58C1(pTiB6S3ΔT)^H^ carrying pBISN1 by the AGROBEST method and analyzed for GUS activity **(A)** and morphologic features **(B)** at different dpi.

Because the highest transient expression efficiency in *efr-1* seedlings can be achieved by infection with C58C1(pTiB6S3ΔT)^H^ by AGROBEST, we chose this combination to test the versatility and applicability of AGROBEST. For example, dissecting the minimal infection time (from 1–5 days) and range of seedling age (from 3- to 6-d-old) applicable for efficient transient expression is of interest. We tested different ages of *Arabidopsis efr-1* seedlings infected at different dpi and noted that GUS signals were barely detectable at 1 dpi but gradually reached a plateau at 3 or 4 dpi (Figure 5A). When 5- or 6-d-old seedlings were infected, we observed transient GUS expression in true leaves at 3 or 4 dpi. Although strong GUS staining could still be detected in seedlings at 4 or 5 dpi, these seedlings often showed bleached lesions in cotyledons (Figure 5B), which explained the lack of GUS expression in part of the cotyledons at 4 or 5 dpi (Figure 5A). At 3 dpi, the bleached lesions were more visible when the transformation was performed with 5- or 6-d-old seedlings than with 3- or 4-d-old seedlings. Thus, the use of younger seedlings for AGROBEST may be more desirable for maintaining plants in healthy and physiological conditions.

**Figure 6:**
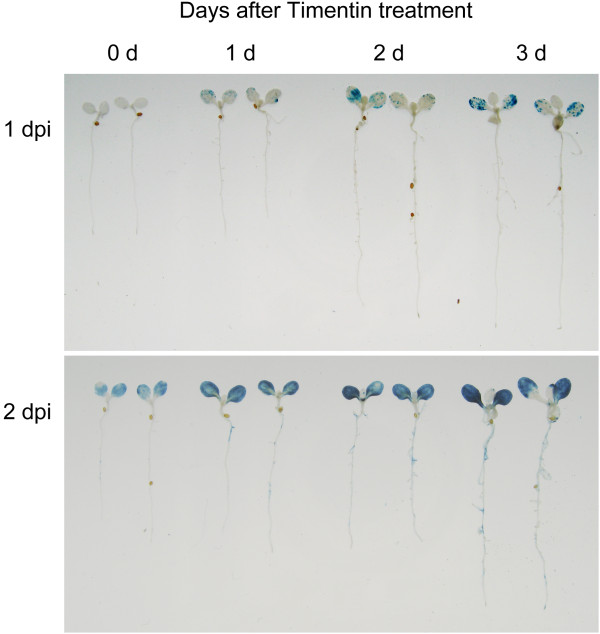
**Impact of Timentin treatment on transient GUS expression efficiency.** Four-day-old *Arabidopsis efr-1* seedlings were infected with *Agrobacterium* C58C1(pTiB6S3ΔT)^H^ carrying pBISN1 by the AGROBEST method at 1 or 2 dpi before Timentin treatment. GUS staining was performed at 0 to 3 days after Timentin treatment.

To test the minimal infection time for GUS detection and to avoid plant damage due to prolonged *Agrobacterium* infection, the co-cultivation medium was replaced with fresh medium containing antibiotics (100 μM Timentin) at 1 or 2 dpi to inhibit bacterial growth. In 4-d-old seedlings, we detected low levels of GUS signals with an additional 2 or 3 days of cultivation after Timentin treatment at 1 dpi (Figure 6). Importantly, with Timentin treatment at 2 dpi, seedlings with 1 to 3 days of additional cultivation remained healthy (without bleached lesions) and showed strong GUS signals in cotyledons. Because *Agrobacterium* cells were mostly killed when true leaves emerged from infected seedlings, the newly grown true leaves were not efficiently transformed. Therefore, the use of 4-d-old seedlings infected for 2 days followed by an additional 1 to 3 days of cultivation with Timentin is the optimal condition to transiently express genes for functional studies.

### Widespread transient transformation events in different organs and cell types

**Figure 7:**
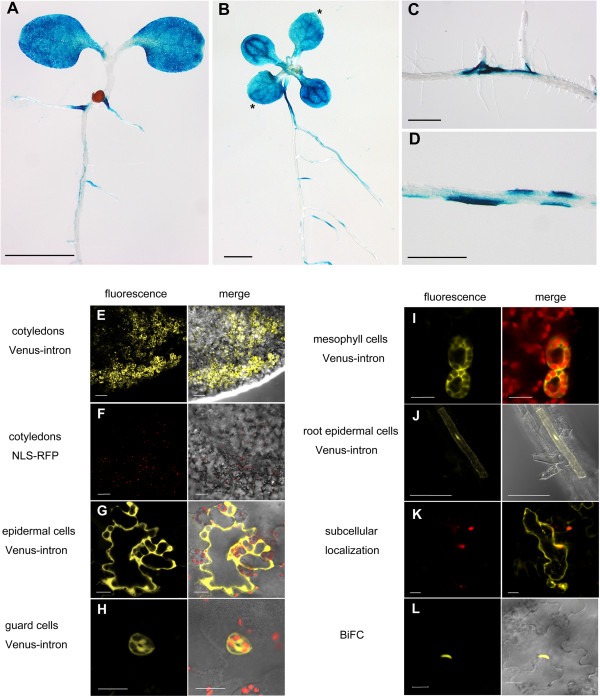
**Transient transformation events in different organs and cell types. (A-D)** Four-day-old **(A and C-D)** or 7-d-old **(B)***Arabidopsis efr-1* seedlings were infected with C58C1(pTiB6S3ΔT)^H^ carrying pBISN1 by the AGROBEST method and analyzed for GUS staining. GUS staining was detected in true leaves (**B**, indicated by asterisk), cotyledons **(A and B)**, main roots near lateral initiation site **(C)**, and elongation zone **(D)**. **(E-L)** Confocal microscopy of 4-day-old *Arabidopsis efr-1* seedlings infected with C58C1(pTiB6S3ΔT)^H^ carrying various vectors for transient expression of indicated fluorescent proteins by the ABM200 method. Fluorescence signals for 35S::Venus-intron or 35S::NLS-RFP were detected in cotyledons **(E and F)**. Venus-intron signals were detected in different types of cells, including epidermal cells **(G)**, guard cells **(H)**, mesophyll cells **(I)** of cotyledon, and root epidermal cells **(J)**. **(K)** Subcellular localization of Venus-intron and NLS-RFP by co-infection of 2 *Agrobacterium* strains expressing 35S::Venus-intron or 35S::NLS-RFP. **(L)** Protein–protein interaction by BiFC of nYFP-ASK1 and TIR1-cYFP. Images show fluorescence alone **(K)** and/or merged with bright field **(E, F, J and L)** or chloroplast fluorescence **(G-I)**. Scale bars are 2 mm **(A and B)**, 0.5 mm **(C and D)**, 100 μm **(E, F and J)**, 50 μm **(L)** and 20 μm **(G-I and K)**. BiFC, bimolecular fluorescence complementation.

The high transient expression efficiency with AGROBEST was mostly evident with strong and homogeneous GUS signals detected in cotyledons of 100% infected Col-0 or *efr-1* seedlings (Figures 3A, 4A and 7A, Additional file 1: Table S1). When 7-d-old seedlings were used for infection, strong GUS signals were also detected in true leaves, as shown in *efr-1* seedlings (Figure 7B). However, in roots, GUS signals could be detected in ~70% of Col-0 or *efr-1* seedlings infected by AGROBEST (Additional file 1: Table S1) and mostly appeared in lateral root initiation sites or in the elongation zone (Figure 7C and D). In addition to analyzing the GUS reporter, we determined the expression of fluorescent proteins as reporters at cellular and subcellular levels using *efr-1* seedlings. With expression of the Venus-intron or NLS-RFP driven by the CaMV 35S promoter, fluorescent protein signals were widely detected in cotyledon cells (Figure 7E and F), mainly in epidermal pavement cells but also in guard cells and mesophyll cells (Figure 7G-I). For roots, epidermal cells consistently showed fluorescent protein signals (Figure 7J). Therefore, the AGROBEST seedling transformation system allows for high transient gene expression and, potentially, functional analysis in diverse tissues and cell types in *Arabidopsis* seedlings.

### Studies of protein subcellular localization and protein–protein interactions

Because *Arabidopsis* plants are less amenable for transient expression analysis, both fluorescent protein localization and bimolecular fluorescence complementation (BiFC) studies are often conducted in protoplasts via transfection or in *N. benthamiana* leaves via agroinfiltration because of the high transient expression efficiency [16,17]. Here, we co-infected two *A. tumefaciens* strains carrying a binary vector for 35S::Venus-intron or 35S::NLS-RFP in *efr-1* seedlings and detected both cytoplasmic and nuclear fluorescence signals for Venus and nuclear localization of NLS-RFP in separate or the same cells (Figure 7K). Our assay is also feasible for BiFC studies, which is supported by the interaction of two known interacting proteins, F-box protein TIR1 (transport inhibitor response 1) and ASK1 (*Arabidopsis* Skp1-like protein) [41], in the nucleus (Figure 7L). Thus, AGROBEST is an ideal system for subcellular localization and protein-protein interaction studies.

### AGROBEST for the expression analysis of a circadian clock reporter gene

**Figure 8:**
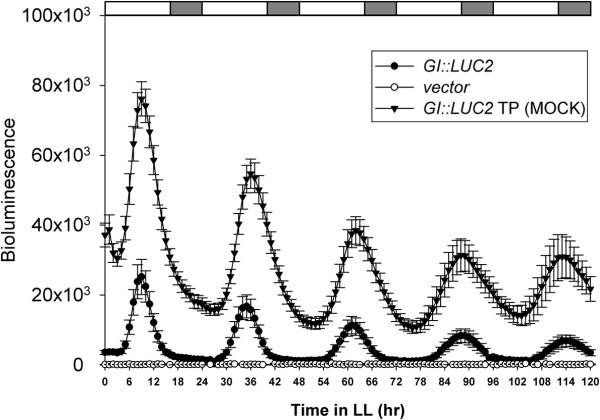
**Monitoring *Arabidopsis *circadian rhythm by transient expression of *GIGANTEA*::luciferase (*GI*::*LUC2*).** Four-day-old *Arabidopsis efr-1* seedlings were infected with *Agrobacterium* C58C1(pTiB6S3ΔT)^H^ carrying a vector (pCAMBIA1390) or p1390-*GI-LUC2* by the AGROBEST method for 3 days in a 16-h/8-h light/dark cycle (75 μmol m^-2^ s^-1^), then transferred to 1/2 MS liquid medium in the presence of 100 μM Timentin and 0.5 mM luciferin and grown under continuous light at 40 μmol m^-2^ s^-1^ for up to 5 days. The *GI*::*LUC2* transgenic *Arabidopsis* plant (TP) cultured in identical conditions without *Agrobacterium* infection was a positive control. Real-time bioluminescence signals were photographed and the luciferase intensity is shown as mean ± SEM from 12 seedlings expressing *GI*::*LUC2*. Similar results were obtained from at least 3 independent experiments. The white and gray regions indicate subjective light and dark periods, respectively.

Encouraged by the high transient expression efficiency with AGROBEST, we next tested its applicability in gene function/regulation study in physiological contexts. Most *Arabidopsis* genes express rhythmically under various thermocycles, photocycles, or circadian clock conditions [42]. Reporter genes driven by promoters of the circadian genes are commonly used to monitor the regulation of circadian genes. To test whether circadian rhythm could be monitored in transiently transformed seedlings, a circadian reporter (*GI::LUC2*) constructed by fusing the promoter of the circadian gene *GIGANTEA* (*GI*) with the luciferase gene (*LUC2*) [43] was used. Four-day-old *Arabidopsis efr-1* seedlings were infected with *Agrobacterium* delivering *GI::LUC2* for 3 days under 16-h/8-h light/dark cycles and then transferred to MS medium in the presence of 100 μM Timentin and 0.5 mM luciferin under continuous light to monitor real-time bioluminescence for 5 days. In contrast to constant low levels of bioluminescence from seedlings infected with a vector control, *Arabidopsis* seedlings infected with *Agrobacterium* delivering *GI::LUC2* showed clear circadian oscillation at slightly lengthened period for at least 5 days (Figure 8). The observed transiently expressed *GI* circadian cycle is indistinguishable from the stable *GI* expression in *GI::LUC2* transgenic *Arabidopsis* plant (TP) [43], although with lower amplitude. The comparable circadian oscillation between the stable and transient expression of the *GI::LUC2* indicated that the slightly longer period we observed was unlikely a result of the *Agrobacterium* infection. This result indicated the applicability of AGROBEST for transient expression of circadian rhythm reporter in *Arabidopsis* seedlings without detectable interference by *Agrobacterium* infection.

### AGROBEST for functional assays of transcription factor MYB75

**Figure 9:**
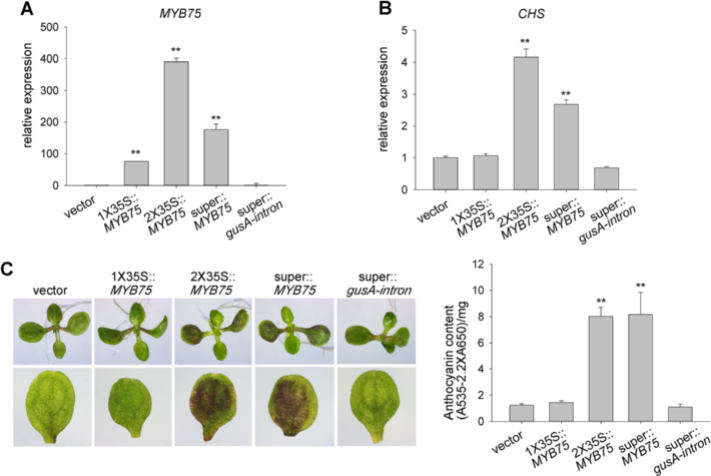
**Transient expression of *MYB75 *increases anthocyanin accumulation.** Four-day-old *Arabidopsis efr-1* seedlings were infected with *Agrobacterium* C58C1(pTiB6S3ΔT)^H^ carrying a vector (pCAMBIA1390), 35S::*MYB75*, 2X35S::*MYB75*, super::*MYB75*, or super::*gusA-intron* by the AGROBEST method. At 3 dpi, co-cultivation medium was replaced with MS medium containing 100 μM Timemtin for additional incubation for 3 days. qRT-PCR of relative expression of *MYB75***(A)** and *CHS***(B)** with representative data shown with mean ± SD from 3 technical repeats. Similar results were obtained from three independent experiments. Zeiss inverted microscopy of anthocyanin accumulation in seedlings (upper panels) and cotyledons (lower panels) and quantification **(C)**. Data for anthocyanin content are mean ± SD from 4 repeats (2 biological repeats from each of 2 independent experiments, 20–30 seedlings for each biological repeat), Values significantly different from that obtained with vector are denoted (**P < 0.01, by Student’s *t* test).

**Figure 10:**
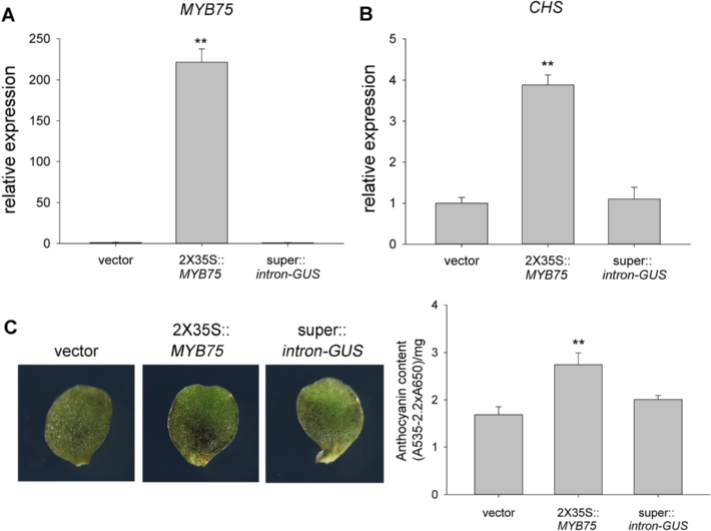
**Transient expression of *MYB75 *increases anthocyanin accumulation in Col-0 seedlings.** Four-day-old *Arabidopsis* Col-0 seedlings were infected with *Agrobacterium* C58C1(pTiB6S3ΔT)^H^ carrying a vector (pCAMBIA1390), 2X35S::*MYB75*, or super::*gusA-intron* by the AGROBEST method. At 3 dpi, co-cultivation medium was replaced with MS medium containing 100 μM timemtin for additional incubation for 3 days. qRT-PCR of relative expression of *MYB75***(A)** and *CHS***(B)** with representative data shown with mean ± SD from 3 technical repeats. Similar results were obtained from three independent experiments. Zeiss inverted microscopy of anthocyanin accumulation in seedlings (upper panels) and cotyledons (lower panels) and quantification **(C)**. Data for anthocyanin content are mean ± SD from 3 independent experiments (20–30 seedlings for each biological repeat, 3 biological repeats for each independent experiment). Values significantly different from those obtained with vector are denoted (**P < 0.01, by Student’s *t* test).

Next, we tested AGROBEST for gain-of-function studies. For a proof of concept, we transiently expressed a transcription factor MYB75 because of its well-established function in anthocyanin accumulation by upregulating a key gene encoding chalcone synthase (CHS) in the anthocyanin synthesis pathway [44]. Four-day-old *efr-1 Arabidopsis* seedlings were infected for 3 days after Timentin treatment for an additional 3 days to determine the effect on *MYB75* transient expression. *MYB75* mRNA level in infected seedlings was 60-, 400-, and 200-fold higher when the expression was driven by single (1X35S) and double (2X35S) CaMV 35S promoter and super promoter, respectively, than in seedlings expressing control vectors (Figure 9A). *CHS* mRNA level was increased 4- and 3-fold in 2X35S::*MYB75* and super::*MYB75* seedlings, respectively (Figure 9B). However, *CHS* expression was not increased in 1X35S::*MYB75* seedlings despite its 60-fold higher *MYB75* expression, which suggests a threshold expression level or the requirement of other MYB75-modulated co-activators for *CHS* activation. Importantly, consistent with increased *CHS* expression, high level of anthocyanin (purple coloration) was readily detectable in cotyledons of 2X35S::*MYB75* and super::*MYB75* seedlings but not in seedlings infected with a vector control, 1X35S::*MYB75*, or super::*gusA*-*intron* (Figure 9C). No increase of anthocyanin accumulation from super::*gusA*-*intron* seedlings indicated that the specificity of the observed anthocyanin phenotype was due to the transient expression of *MYB75* rather than a secondary effect from the infection or the overexpression of any foreign protein. Importantly, AGROBEST also enables the transient expression of *MYB75* to monitor its downstream *CHS* expression and anthocyanin accumulation in Col-0 seedlings. We show that transient expression of *MYB75* driven by the 2X35S promoter results in significantly higher of *MYB75* mRNA levels than vector control in Col-0 seedlings (Figure 10A). Remarkably, *CHS* mRNA levels were also upregulated in 2X35S::*MYB75* seedlings (Figure 10B), in which a moderate increase in anthocyanin accumulation was also detected (Figure 10C). This result strongly suggested the broad application of AGROBEST for gain-of-function studies not limited to the immune-compromised mutant.

## Discussion

### AGROBEST enables high transient transformation and expression efficiency in intact *Arabidopsis* young seedlings

In this study, we developed a simple, fast, reliable, and robust transient expression system named AGROBEST and uncovered the key factors enabling 100% of infected seedlings with high transgene expression efficiency in *Arabidopsis* seedlings. Remarkably, AGROBEST appears to achieve the highest transient expression efficiency in the EF-Tu receptor *efr-1* mutant as compared to the wild-type Col-0, flagellin receptor mutant *fls2*, and DEX-inducible AvrPto transgenic line. This result is consistent with a previous finding in agroinfiltrated *Arabidopsis* adult leaves showing increased transient GUS expression efficiency in *efr-1*[24]. Because of no detectable phenotype impairing the growth and development in the *efr* mutant [24], the use of the *efr* mutant has an advantage over DEX-inducible AvrPto in seedling stages. Thus, more selected elimination of specific PRRs such as EFR with minimal effects on hormonal signaling, cell death and seedling growth may be a preferred system for *Agrobacterium-*mediated high transient expression efficiency. Interestingly, *N. benthamiana* leaves, which are commonly used for *Agrobacerium*-mediated transient transformation, also lack the EFR receptor [45].

Unexpectedly, we discovered that AGROBEST also enables high transient expression efficiency in wild-type Col-0 seedlings. The significantly higher transient expression activity by AGROBEST than the FAST and Marion et al. methods likely accounts for the success of our gain-of-function experiments, which have not been shown previously [26,27]. Of note, *efr-1* seedlings remained poorly transformed by FAST method as compared with the significantly increased transient expression in *efr-1* by AGROBEST or the Marion et al. method. The reason underlying this discrepancy is unknown, but the yellowish and retarded-growth seedlings after co-cultivation with *Agrobacterium* in the dark for 2 days by the FAST method may contribute to the observed phenotype. Our AGROBEST method, applying a lower density of *Agrobacterium* cells (OD_600_ 0.02 as opposed to OD_600_ 0.5 for the FAST method and OD_600_ 2 for the Marion et al. method) for co-cultivation with seedlings without any mechanical treatment (e.g., vacuum infiltration) or chemical treatment (e.g., the addition of surfactant Silwet L-77) offers advantages to maintain infected seedlings with normal growth and a physiological state without injury. The success of transiently expressing the circadian rhythm reporter in *Arabidopsis* seedlings may open a new platform to rapidly test the circadian behaviors of *Arabidopsis* mutants, bypassing the process of introducing a circadian reporter gene into the mutants by crossing. Most remarkably, AGROBEST allows for high transient expression of the *MYB75* transcription factor and subsequently upregulates the expression of its downstream gene *CHS* in both *efr-1* and Col-0 seedlings. This result suggested the broad application of AGROBEST to study transcription factor action.

### Widespread and differential transient transformation events in different organs and cell types

AGROBEST has a breakthrough performance by enabling high and homogeneous transient GUS expression efficiency in shoots of 100% infected Col-0 or *efr-1* seedlings. The successful transient expression in roots, although with less efficient transformation events (~70% of seedlings with GUS staining in roots), is also remarkable and not previously detected [26,27]. Interestingly, preferential transformation events occurring at the initiation sites of lateral roots or the root elongation zone of infected intact seedlings were also previously detected in wounded *Arabidopsis* roots [46]. High transformation of *Arabidopsis* roots may require further loosening or opening of cell walls or wounding, which was not included in our infection conditions. Because we observed similar transient expression levels and transformation efficiency in roots of Col-0 and *efr-1* seedlings (Additional file 1: Table S1), EFR may play no or little role in seedling root transformation efficiency under our infection conditions. Consistently, *EFR* is expressed at low levels in Col-0 seedling roots [47], which were not responsive to the EF-Tu peptide elf26, as evidenced by limited induction of immune marker genes and callose deposition in the roots of Col-0 seedlings [48]. Because the flg22 peptide derived from *Agrobacterium* flagellin is inactive in *Arabidopsis*[24,34,35] and the flagellin receptor mutant exhibited similar transformation efficiency as Col-0 in our seedling assays, the flagellin receptor FLS2 may not be involved in *Agrobacterium*-triggered plant innate immune responses and therefore did not compromise *Agrobacterium*-mediated transient gene expression. Future investigations could examine whether the absence of the peptidoglycan receptor [49] or yet-to-be identified receptors in recognizing additional MAMPs such as polysaccharides [50] could increase the transformation efficiency in seedling roots.

### Key factors for high transient transformation/expression efficiency

During this course of our method development, we also uncovered new factors critical for the high transient transformation/expression efficiency in *Arabidopsis* seedlings. One factor is the addition of AB salts in MS medium buffered with acidic pH 5.5 during *Agrobacterium* infection, which allows for significantly higher transient expression efficiency than in MS medium alone. Another breakthrough is the use of the disarmed *A. tumefaciens* strain C58C1(pTiB6S3ΔT)^H^, which offers the highest transient expression efficiency with the least adverse impact on plant growth over other tested strains. Root growth was severely inhibited on infection with other tested *A. tumefaciens* strains including the transfer-incompetent Δ*virB2*. These data indicate that the transport of T-DNA and T4SS effectors into plant cells by a virulent C58 strain may not suppress host immune responses like that observed in T3SS effectors from *Pseudomonas syringae*[51]. We observed that C58C1(pTiB6S3ΔT)^H^ achieved higher transient expression efficiency in both Col-0 and *efr-1* seedlings than other *A. tumefaciens* strains tested. The agent also had little impact on root growth inhibition of infected seedlings by the ABM50 method (Figure 3). The results suggested that the *A. tumefaciens* strain C58C1(pTiB6S3ΔT)^H^ is the main factor affecting the root growth difference. EFR may play a minor role in root growth inhibition because we observed slightly stronger root growth inhibition in Col-0 than *efr-1* seedlings infected with C58C1(pTiB6S3ΔT)^H^. This finding is consistent with limited root growth inhibition detected in Col-0 seedlings in response to EF-Tu peptide elf18 as compared with strong root growth inhibition induced by flg22 [47]. The observed inverse association of root growth inhibition and transient expression efficiency suggested that C58C1(pTiB6S3ΔT)^H^ may circumvent a plant defense barrier to enable high transient expression levels in *Arabidopsis* seedlings. However, interestingly, root length was significantly lower in Col-0 and *efr-1* seedlings with C58C1(pTiB6S3ΔT)^H^ infection than in uninfected seedlings (MOCK), despite the significantly higher transient expression efficiency with the AGROBEST than the ABM50 method (Figure 3). Thus, although C58C1(pTiB6S3ΔT)^H^ remains a strain causing the least inhibition in seedling root growth as compared to other *A. tumefaciens* strains, whether the observed root growth inhibition results from PTI contributing to reduce transient expression efficiency requires future investigation. Other factors in addition to PTI may contribute to the enhanced transient expression efficiency by AGROBEST.

C58C1(pTiB6S3ΔT)^H^ has been known to achieve high transformation efficiency in several plant species including *Arabidopsis*, but the underlying mechanism is not known. The nomenclature of *Agrobacterium* strains used in plant transformation experiments is often simplified, which causes confusion and could sometimes be misleading. C58C1(pTiB6S3ΔT)^H^ is often simplified as C58C1 in the plant community. C58C1 is in fact named after curing pTiC58 from the wild-type virulent strain C58, and rifampicin (Rif)-resistant strains are selected from C58C1 for convenient use to acquire various disarmed Ti plasmids transferred from different *Agrobacterium* strains [52,53]. Therefore, C58C1(pTiB6S3ΔT)^H^ is a Rif-resistant C58C1 harboring the octopine-type Ti plasmid pTiB6S3 with the removal of the T-DNA region [31] and containing a pCH32 helper plasmid with increased expression of virulence genes *virG* and *virE2*[32]. GV3101(pMP90) is a C58-derived disarmed strain, in which pMP90 is a nopaline-type Ti plasmid, pTiC58, with the removal of T-DNA [38]. Therefore, in theory, C58C1(pTiB6S3ΔT)^H^ should share the same chromosomal background with GV3101(pMP90) and only differ in the use of different Ti plasmids and the presence of the helper plasmid pCH32. Future work to determine which genetic factor(s) contribute to increased transient expression efficiency with less growth inhibition by C58C1(pTiB6S3ΔT)^H^ will shed light on understanding the molecular mechanisms underlying the observed high transient transformation and expression efficiency.

## Conclusions

In this study, we developed a valuable and novel method, named AGROBEST, and uncovered the key factors enabling this unprecedented high transient transformation and reporter gene expression efficiency in the immune receptor mutant *efr-1* and in wild-type Col-0 *Arabidopsis* seedlings. The applicability for transient expression of *MYB75* in activating downstream gene expression in a Col-0 background further suggested that AGROBEST may be a feasible method to use in examining transcription factor actions or gain-of-function studies in different *Arabidopsis* ecotypes/genotypes. Because most plants do not harbor *EFR*, which is only present in *Brassicaceae*[24], the established method may be applicable in other plant species. This fast, sensitive, and quantitative assay was routinely used with culture plates, which are easily scaled up for quick and systematic screens. Importantly, this method nicely compliments the commonly used *Arabidopsis* mesophyll-protoplast transfection [15,16] and *Agrobacterium-*mediated leaf infiltration in *N. benthamiana*[17] for gene functional studies and provides advantages for its high reproducibility without advanced skills. Furthermore, AGROBEST may be an alternative method for evaluating *Agrobacterium* virulence and discovering and dissecting gene functions involved in various steps of *Agrobacterium*-mediated DNA transfer. The method may help unravel the mechanisms underlying *Agrobacterium* infection in unwounded cells/tissues.

## Methods

### Materials and growth condition

Strains, plasmids, and primer sequences used in this study are in Additional file 2: Table S2 and Additional file 3: Table S3. The bacterial growth conditions and procedures for plasmid and mutant constructions are described in Additional file 4: Methods S1. *Arabidopsis thaliana* plants included ecotype Columbia-0 (Col-0), Wassilewskija (Ws-2), T-DNA insertion mutants *efr-*1 (SALK_044334) and *fls2* (SALK_093905) and the DEX-inducible AvrPto transgenic line generated in a Col-0 background were obtained from the Arabidopsis Biological Resource Center (Ohio). Seeds were sterilized in 50% bleach (v/v) containing 0.05% Triton X-100 (v/v) for 10 min, rinsed 5 times with sterile water, and incubated at 4°C for 3 days. For germination, 10 seeds were transferred to 1 ml 1/2 MS liquid medium (1/2 MS salt supplemented with 0.5% sucrose (w/v), pH 5.5 [pH adjusted to 5.7 by KOH but pH 5.5 after autoclaving], in each well of a 6-well plate. Germination and growth took place in a growth room at 22°C under a 16-hr/8-hr light–dark cycle (75 μmol m^-2^ s^-1^).

### *Agrobacterium* infection in *Arabidopsis* seedlings

For AGROBEST infection assay, *A. tumefaciens* was freshly streaked out from -80°C glycerol stock onto a 523 agar plate for 2-day incubation at 28°C. A fresh single colony from the plate was used to inoculate 5 ml of 523 liquid medium containing appropriate antibiotics for shaking (220 rpm) at 28°C for 20–24 hr. For pre-induction of *A. tumefaciens vir* gene expression, *A. tumefaciens* cells were pelleted and re-suspended to OD_600_ 0.2 in various liquid media including LB, LB-MES (LB with 10 mM MES, pH 5.7) [53,54] or AB-MES (17.2 mM K_2_HPO_4_, 8.3 mM NaH_2_PO_4_, 18.7 mM NH_4_Cl, 2 mM KCl, 1.25 mM MgSO_4_, 100 μM CaCl_2_, 10 μM FeSO_4_, 50 mM MES, 2% glucose (w/v), pH 5.5) [37] with different concentrations of acetosyringone (AS; 0, 50 or 200 μM) without antibiotics, then shaken (220 rpm) at 28°C for 12–16 hr. Before infection, *A. tumefaciens* cells were pelleted and re-suspended in desired co-cultivation liquid media to OD_600_ 0.02. The growth medium of *Arabidopsis* seedlings was replaced with 1 ml *A. tumefaciens* cells freshly prepared above and incubated in the same growth room until further analysis. Three-day-old seedlings were treated with 10 μM DEX for 1 day before infection for the following 3 days. When the removal of *Agrobacterium* cells was required, co-cultivation medium was removed after the chosen infection time and replaced with 1 ml freshly prepared MS medium containing 100 μM Timentin and incubated for additional days before analysis. The procedures for the seedling transient transformation assay using the method optimized by Marion et al. and FAST Method developed by Li et al. were performed [26,27] and described in Additional file 4: Methods S1. Unless indicated, 10 seedlings grown in each well were infected and 3 biological repeats were performed in each independent experiment.

### Plant RNA extraction and quantitative RT-PCR

RNA was extracted from *Arabidopsis* seedlings as described [55]. An amount of 4 μg total RNA was used to synthesize first-strand cDNA with SuperScript III Reverse Transcriptase (Invitrogen) and oligo dT primer. Quantitative PCR involved the Applied Biosystems QuantStudio 12 K Flex Real Time PCR machine and Power SYBRR Green PCR Master Mix (Invitrogen). *Arabidopsis ACTIN 2* (At3g18780) or *UBC21* (At5g25760) was an internal control.

### GUS staining and activity assays

Seedlings were stained with 5-bromo-4-chloro-3-indolyl glucuronide (X-Gluc) at 37°C for 6 hr unless indicated or quantified with a fluorescence substrate (4-methylumbelliferyl-β-D-glucuronide [MUG]) as described [56]. For MUG assay, fluorescence was determined using a 96 microtiter-plate reader (Bio-Tek Synergy Mx, 356 nm excitation 455 nm emission with ±20 nm filter) and calculation of specific GUS enzyme activity was based on the standard curve of 0.5–500 pmole (0.5, 5, 50 and 500 pmole) 4-MU standards obtained from the same microtiter plate. For relative GUS activity, the fluorescence signal value was normalized by an equal amount of proteins with subtraction of the background fluorescence signal detected by the mock control.

### Confocal microscopy

Fluorescence signals were observed by use of a Zeiss LSM 510 Meta Confocal microscope. Venus signals were observed at 488-nm excitation with an HFT 488/514-nm filter and emission with NTF 515- and BP 505- to 530-nm filters. RFP signals were observed at 488-nm excitation with an HFT 405/488-nm filter and emission with NFT 545 and LP 650 filters.

### Transient expression of *MYB75* and anthocyanin content assay

Four-day-old seedlings were infected with *A. tumefaciens* strain C58C1(pTiB6S3ΔT)^H^ carrying the control or *MYB75*-expressing binary vector in ABM-MS liquid medium for 3 days. The co-cultivation medium was then replaced with 1 ml fresh MS medium (1/2 MS, 2% sucrose (w/v), pH adjusted to 5.7 by KOH but pH 5.5 after autoclaving) containing 100 μM Timemtin and then incubated for 3 days. For anthocyanin content assay, seedlings were blot-dried briefly, weighed, ground into powder with liquid nitrogen and mixed with 1 ml extraction buffer (0.12 M HCl, 18% isopropanol (v/v)). The mixture was boiled for 90 sec and centrifuged at 16000 × g for 15 min. The supernatant was collected and measured at OD_535_ (A535) and OD_650_ (A650). Anthocyanin content was calculated as A535 - (2.2 × A650)/fresh weight (g) [57].

### Transient expression of *GI::LUC2* and bioluminescence measurement

Four-day-old seedlings were infected with *A. tumefaciens* strain C58C1(pTiB6S3ΔT)^H^ carrying p1390-*GI::LUC2* or empty vector (pCAMBIA1390) in ABM-MS co-cultivation medium. At 3 dpi, each seedling was transferred to MS medium (1/2 MS, pH adjusted to 5.7 by KOH but pH 5.5 after autoclaving) containing 100 μM Timentin and 0.5 mM luciferin in a black 96-well plate. Bioluminescence activity was measured and analyzed as described [43].

### Luciferase activity assay

*Arabidopsis* seedlings after infection were surface sterilized with 1% bleach (0.05% sodium hypochlorite) for 5–10 min and washed with sterile water 3 times to remove bacteria before assay. The washing step is essential to minimize the background signals expressed in bacteria because of the use of intron-less *LUC2* reporter. For photography, 10 seedlings infected by each method were placed in a clean 15-cm square Petri dish and covered with 100 μl 1 mM luciferin. Luciferase intensity was imaged by use of the XENOGEN IVIS lumina system with 5-sec exposure time. Bioluminescence assay involved the luciferase assay system (Promega). Briefly, 10–15 seedlings after a washing were blot-dried with tissue paper before being frozen with liquid nitrogen and stored at -80°C. Seedlings were ground into fine powder by liquid nitrogen, mixed with 300 μl cell-culture lysis reagent (Promega), and centrifuged at 16000 × g for 10 min at 4°C. Supernatant was 100× diluted with cell-culture lysis reagent. In total, 20 μl cell lysate was mixed with 100 μl Luciferase Assay Reagent and the signal was detected by use of lumat LB 9507 (Berthold Technologies). The bioluminescence signal was normalized to the protein amount of each sample quantified by the Bradford protein assay (Bio-Rad).

## Competing interests

The authors declare that they have no competing interests.

## Authors’ contributions

HYW participated the experimental designs, performed most of the experiments, analyzed data, and drafted the manuscript. KHL and WLC participated in method optimization. YCW and JFW performed experiments. CYC participated in data analysis. SHW and JS participated in experimental designs and helped drafting the manuscript. EML conceived of the study, participated in method optimization and experimental designs, coordinated the project, and wrote the manuscript. All authors read and approved the final manuscript.

## Supplementary Material

Additional file 1: Table S1 Transient transformation efficiency of shoots and roots of *Arabidopsis* Col-0 and *efr-1* seedlings. Click here for file

Additional file 2: Table S2 Bacterial strains and plasmids. Click here for file

Additional file 3: Table S3 Primer information. Click here for file

Additional file 4: Methods S1 Methods for bacterial strains and plasmids and infection method by Li et al. (FAST method) and by Marion et al. Click here for file
