# Supplementary material for: AGROBEST: an efficient Agrobacterium-mediated transient expression method for versatile gene function analyses in Arabidopsis seedlings
Source: Plant Methods. 2014 Jun 18;10:19. doi: 10.1186/1746-4811-10-19 (PMC4076510; doi:10.1186/1746-4811-10-19)
Supplement: Additional file 1: Table S1 — Transient transformation efficiency of shoots and roots of Arabidopsis Col-0 and efr-1 seedlings. [file 1746-4811-10-19-S1.docx]

**Table S1** Transient transformation efficiency of shoots and roots of *Arabidopsis* Col-0 and *efr-1* seedlings.

| Genotype | Method | Organ | Repeat^a^ | | | | | | | | |
| --- | --- | --- | --- | --- | --- | --- | --- | --- | --- | --- | --- |
|  |  |  | 1 | 2 | 3 | 4 | 5 | 6 | 7 | 8 | Sum (%) |
| Col-0 | ABM50 | Shoot | 11/11 | 10/10 | 9/9 | 11/11 | NA | NA | NA | NA | 41/41 (100%) |
|  |  | Root | 1/11 | 2/10 | 1/9 | 0/11 | NA | NA | NA | NA | 4/41 (9.8%) |
| Col-0 | AGROBEST | Shoot | 12/12 | 10/10 | 10/10 | 11/11 | NA | NA | NA | NA | 43/43 (100%) |
|  |  | Root | 9/12 | 6/10 | 8/10 | 6/11 | NA | NA | NA | NA | 29/43 (67.4%) |
| *efr-1* | ABM50 | Shoot | 10/10 | 12/12 | 9/9 | 9/9 | 9/9 | 8/8 | 9/9 | 9/9 | 75/75 (100%) |
|  |  | Root | 8/10 | 4/12 | 5/9 | 3/9 | 7/9 | 5/8 | 6/9 | 3/9 | 41/75 (54.7%) |
| *efr-1* | AGROBEST | Shoot | 12/12 | 11/11 | 9/9 | 11/11 | 8/8 | 10/10 | 10/10 | 10/10 | 81/81 (100%) |
|  |  | Root | 9/12 | 9/11 | 7/9 | 9/11 | 6/8 | 5/10 | 6/10 | 7/10 | 58/81 (71.6%) |

^a^Transient transformation efficiency was determined by counting all seedlings (~10) in each well (biological repeat) for the detection of homogeneous GUS strains in shoots or roots of Col-0 and *efr-1* seedlings infected with C58C1(pTiB6S3ΔT)^H^ by the indicated methods. Data are number of seedlings with GUS staining/number of infected seedlings. NA, not available.
