# Supplementary material for: AGROBEST: an efficient Agrobacterium-mediated transient expression method for versatile gene function analyses in Arabidopsis seedlings
Source: Plant Methods. 2014 Jun 18;10:19. doi: 10.1186/1746-4811-10-19 (PMC4076510; doi:10.1186/1746-4811-10-19)
Supplement: Additional file 2: Table S2 — Bacterial strains and plasmids. [file 1746-4811-10-19-S2.pdf]

Table S2 Bacterial strains and plasmids

Strains / plasmids	Relevant characteristics	Source/reference
A. tumefaciens strains		
C58	Wild type virulent strain	[1]
C58C1	C58C1, which is C58 cured pTiC58	[2]
C58C1(pTiB6S3ΔT)^{H}	$Rm^R Cb^R Tc^R$, Rm^R strain of C58C1 harboring pTiB6S3ΔT-DNA and helper plasmid pCH32	[3]
GV3101(pMP90)	$Rm^R Gm^R$, Rm^R strain of C58C1 harboring pTiC58ΔT-DNA	[4]
C58Δ*virB2*	*virB2* in-frame deletion mutant in C58	This study
Plasmids		
pJQ200KS	Gm^R, suicide plasmid containing Gm^R and *sacB* gene for selection of double crossover	[5]
pJQ-VirB2	Gm^R, pJQ200KS-derived plasmid used to generate *virB2* in-frame deletion mutant	This study
p326-His	Ap^R cloning vector derived from 326GFP [6]	This study
NLS-RFP	Ap^R Cloning vector contain NLS::RFP construct	[6, 7]
pSAT1-nEYFP-C1	Ap^R, BiFC vector	[8]
pSAT1-nEYFP-TIR1	Ap^R, nEYFP-TIR1 driven by 35S promoter for BiFC	[9]
pSAT4-cEYFP-C1-B	Ap^R, BiFC vector	[8]
pSAT4-cYFP-ASK1	Ap^R, cYFP-ASK1 driven by 35S promoter for BiFC	[9]
pBISN1	Km^R, Binary vector expressing *gusA-intron* driven by super promoter ,	[10]
pCAMBIA0390	Km^R, Binary vector for cloning	Cambia

pCAMBIA1390	Km^R, Binary vector for cloning	Cambia
pE3730	Sp^R, Binary vector expressing Venus-intron driven by 35S promoter	Lan-Ying Lee and Stanton Gelvin
pCAMBIA1390-NLS-RFP	Km^R, 35S promoter driven NLS-RFP in pCAMBIA1390	This study
pCAMBIA1390-1X35S-MYB75	Km^R, 1X35S promoter driven *MYB75* in pCAMBIA1390	This study
pCAMBIA1390-2X35S-MYB75	Km^R, 2X35S promoter driven *MYB75* in pCAMBIA1390	This study
pCAMBIA1390-super-MYB75	Km^R, Super promoter driven *MYB75* in pCAMBIA1390	This study
pCAMBIA1390-GI-LUC2	Km^R, GI promoter driven luciferase in pCAMBIA1390	[7]
pCAMBIA1390-1X35S-LUC2	Km^R, 1X35S promoter driven *LUC2* in pCAMBIA1390	This study
pCAMBIA0390-TIR1	Km^R, 35S promoter driven nEYFP-TIR1 in pCAMBIA1390	This study
pCAMBIA0390-SAT1-nEYFP-C1	Km^R, BiFC binary vector expressing nEYFP	This study
pCAMBIA0390-ASK1	Km^R, 35S promoter driven cYFP-ASK1 in pCAMBIA1390	This study
pCAMBIA0390-SAT4-cEYFP-C1-B	Km^R, BiFC binary vector expressing cEYFP	This study

References

1. Hamilton RH, Fall MZ: **The loss of tumor-initiating ability in *Agrobacterium tumefaciens* by incubation at high temperature.** *Experientia* 1971, **27:**229-230.
2. Van Larebeke N, Engler G, Holsters M, Van den Elsacker S, Zaenen I, Schilperoort RA, Schell J: **Large plasmid in *Agrobacterium tumefaciens* essential for crown gall-inducing ability.** *Nature* 1974, **252:**169-170.
3. Deblaere R, Bytebier B, De Greve H, Deboeck F, Schell J, Van Montagu M, Leemans J: **Efficient octopine Ti plasmid-derived vectors for *Agrobacterium*-mediated gene transfer to plants.** *Nucleic acids Res* 1985,

13:4777-4788.

4. Koncz C, Schell J: **The promoter of TL-DNA gene 5 controls the tissue-specific expression of chimaeric genes carried by a novel type of *Agrobacterium* binary vector.** *Mol Gene Genomics* 1986, **204:**383-396.
5. Quandt J, Hynes MF: **Versatile suicide vectors which allow direct selection for gene replacement in gram-negative bacteria.** *Gene* 1993, **127:**15-21.
6. Lee YJ, Kim DH, Kim YW, Hwang I: **Identification of a signal that distinguishes between the chloroplast outer envelope membrane and the endomembrane system in vivo.** *Plant Cell* 2001, **13:**2175-2190.
7. Wang Y, Wu JF, Nakamichi N, Sakakibara H, Nam HG, Wu SH: **LIGHT-REGULATED WD1 and PSEUDO-RESPONSE REGULATOR9 form a positive feedback regulatory loop in the *Arabidopsis* circadian clock.** *Plant Cell* 2011, **23:**486-498.
8. Citovsky V, Lee LY, Vyas S, Glick E, Chen MH, Vainstein A, Gafni Y, Gelvin SB, Tzfira T: **Subcellular localization of interacting proteins by bimolecular fluorescence complementation *in planta*.** *J Mol Biol* 2006, **362:**1120-1131.
9. Chen CC, Liang CS, Kao AL, Yang CC: **HHP1, a novel signalling component in the cross-talk between the cold and osmotic signalling pathways in *Arabidopsis*.** *J Exp Bot* 2010, **61:**3305-3320.
10. Narasimhulu SB, Deng XB, Sarria R, Gelvin SB: **Early transcription of *Agrobacterium* T-DNA genes in tobacco and maize.** *Plant Cell* 1996, **8:**873-886.
