# Supplementary material for: AGROBEST: an efficient Agrobacterium-mediated transient expression method for versatile gene function analyses in Arabidopsis seedlings
Source: Plant Methods. 2014 Jun 18;10:19. doi: 10.1186/1746-4811-10-19 (PMC4076510; doi:10.1186/1746-4811-10-19)
Supplement: Additional file 3: Table S3 — Primer information. [file 1746-4811-10-19-S3.pdf]

**Table S3** Primer information

| Primer name | Sequence (5 '-3') | Annotation |
|---|---|---|
| 326-His-S | GATCCGGACCCGGGGGACTCGAGCAC CACCACCACCACCACTAAGAGCT | Annealing products was ligated into 326GFP at BamHI-SacI sites to replace GFP and generate p326-His |
| 326-His-AS | CTTAGTGGTGGTGGTGGTGGTGCTCGA GTCCCCCGGGTCCG | |
| MYB75(at1g56650)-XbaI-S | CGTCTAGAATGGAGGGTTCGTCCAAA | To amplify *MYB75* CDS |
| MYB75(at1g56650)-XhoI-AS | GCCTCGAGATCAAATTTCACAGTCTC | |
| pSAT4-2X35S-PstI-S | ATTCTGCAGTTTCCCAGTCACGACGTT GT | To amplify 2X35S promoter sequence |
| pSAT4-2X35S-XbaI-AS | TGCTCTAGACTATCGTTCGTAAATGGT GA | |
| VirB2-up-SacI-sense | TTAAGAGCTCGCAGAACCAGGCTCAA GC | For generate *virB2* upstream DNA fragment for pJQ-virB2 |
| VirB2-up-SpeI-anti | TCTACTAGTGCATCGCATTATTGCGGA CCT | |
| VirB2-down-SpeI-sense | TAATACTAGTGGCAAAACGCTGACTGG AGG | For generate *virB2* downstream DNA fragment for pJQ-virB2 |
| VirB2-dwon-XhoI-anti | TAAGCTCGAGGTCATTGCGAAGGAGTT T | |
| pSATN-SpeI-sense | TTACACTAGTGGTTTTCCCAGTCACGA CG | For cloning BiFC expression cassette |
| pSATN-SpeI-anti | TAACAACTAGTCACAGGAAACAGCTAT GACC | |
| NLSRFP-XbaI-sense | AGATTCTAGAACTAGTGGATCCCCCGG GC | For amplify NLS-RFP cassette |
| NLSRFP-SacI-anti | ACTTGAGCTCACGATTCTACAGGAACA GG | |
| Actin 2F-qPCR | ATCGGTGGTTCCATTCTTGCT | qPCR primers for ACT2 (Atg18780) |
| Actin 2R-qPCR | GATTCCTGGACCTGCCTCATC | |
| UBC21-S | TTCAAATGGACCGCTCTTATCA | qPCR primers for *UBC21* (At5g25760) |
| UBC21-AS | AAACACCGCCTTCGTAAGGA | |
| MYB75-157-ABI-S | CCTGTAAGAGCTGGGCTAAACC | qPCR primers for *MYB75* (At1g56650) |
| MYB75-306-ABI-AS | AGACCACCTATTCCCTAGAAGCC | |
| CHS-ABI-S | GTTTCGGACCAGGTCTCACTGT | qPCR primers for *CHS* |

| | | |
|---|---|---|
| CHS-ABI-AS | AGAGAGGAACGCTGTGCAAGA | (At5g13930) |
