# Supplementary material for: AGROBEST: an efficient Agrobacterium-mediated transient expression method for versatile gene function analyses in Arabidopsis seedlings
Source: Plant Methods. 2014 Jun 18;10:19. doi: 10.1186/1746-4811-10-19 (PMC4076510; doi:10.1186/1746-4811-10-19)
Supplement: Additional file 4: Methods S1 — Methods for bacterial strains and plasmids and infection method by Li et al. (FAST method) and by Marion et al. [file 1746-4811-10-19-S4.pdf]

**Methods S1**

**Bacterial strains and plasmids**

*A. tumefaciens* and *E. coli* were grown in 523 medium [1] at 28°C and LB at 37°C, respectively, with appropriate antibiotics. The plasmids were maintained with the addition of appropriate antibiotics (see Additional file 2: Table S2) at the following concentrations: 50 μg/ml gentamycin (Gm), 10 μg/ml tetracyclin (Tc), 50 μg/ml carbenicillin (Cb), 150 μg/ml spectinomycin (Sp), and 20 μg/ml kanamycin (Km). The *MYB75* coding sequence was PCR-amplified and ligated into a p326-His vector at XbaI-XhoI sites to generate the 35S::*MYB75* expression cassette, which was then inserted into pCAMBIA1390 (Cambia) at Pst1-EcoR1 sites to generate p1390-35S-MYB75 for expressing 6xHis-tagged MYB75 driven by a CaMV 35S promoter. To replace the single 35S (1X35S) promoter on p1390-35S-MYB75 with a double 35S (2X35S) promoter, the 2X35S promoter sequence on pSAT4-cEYFP-N1 [2] was PCR-amplified and inserted into p1390-35S-MYB75 at Pst1-XbaI sites. For expression of *MYB75* by a super promoter, *MYB75* CDS was cut out from p1390-35S-MYB75 and inserted into pBISN1 at XbaI-SacI sites, replacing the original *gusA-intron* gene. To construct p1390-NLS-RFP, the NLS-RFP cassette was PCR-amplified and ligated into pCAMBIA1390 at XbaI-SacI sites. To generate binary vectors for BiFC, each expression cassette was amplified from respective pSAT vectors and ligated into pCAMBIA0390 at the SpeI site. To construct pCAMBIA1390-1X35S-LUC2 for expression of *LUC2* by 35S promoter, the 35S promoter digested from p1390-35S-MYB75 was inserted at SalI-PstI sites of pCAMBIA1390-GI-LUC2 to replace the *GI* promoter. The *virB2* in-frame deletion mutant was generated by use of a markerless gene replacement method with the suicide plasmid pJQ200KS [3]. The upstream (550-bp) and downstream (720-bp) segments of *virB2* were PCR-amplified, digested with SacI-SpeI and SpeI-XhoI,

respectively, and inserted into pJQ200KS at SacI-XhoI sites to generate the plasmid pJQ-*virB2.* Subsequently, pJQ-*virB2* was used to generate a *virB2* in-frame deletion mutant in the *A. tumefaciens* strain C58 as described [4]. All primers used are in Additional file 3: Table S3.

**Infection procedures by Li et al. (FAST method)**

The FAST method developed by Li et al. was performed [5] and described as follows**.** *Arabidopsis* seeds were sterilized and sown on 1/4 MS agar (Phytoblend, Caisson Laboratories) plate with 1% sucrose, pH 6.0 and incubated in a 16-hr (22°C)/8-hr (18°C) light-dark cycle. *A. tumefaciens* was freshly streaked out from -80°C glycerol stock onto an agar plate with proper antibiotics for 2 days at 28°C. A fresh single colony from the plate was used to inoculate LB liquid medium containing appropriate antibiotics with shaking (220 rpm) at 28°C for 18-24 hr. Bacterial suspensions were diluted into fresh YEB liquid medium (5 g/L beef extract, 1 g/L yeast extract, 5 g/L peptone, 5 g/L sucrose, 0.5 g/L $MgCl_2$) at $OD_{600}$=0.3 and further grown for 4-6 hr until $OD_{600}$ 1.5-2.0. Bacteria cells were harvested by centrifugation at 6,000 g for 5 min and washed once with washing solution (10 mM $MgCl_2$, 100 μM acetosyringone) and re-suspended in 1 ml washing solution. Four-day-old *Arabidopsis* seedlings were soaked with 20 ml co-cultivation medium (1/4 MS, 1% sucrose, pH 6.0, 100 μM AS, 0.005% Silwet L-77) with *Agrobacterium* suspension at $OD_{600}$ 0.5 in a clean 9-cm Petri dish. Co-cultivation was performed for 36-40 hr in the dark before GUS or luciferase activity assay.

**Infection procedures by Marion et al.**

The optimized infection method developed by Marion et al. was performed [6] and described as follows**.** *Arabidopsis* seeds were sterilized and sown on *Arabidopsis* MS

phyto agar (Duchefa) in six-well plates and incubated in a 16-hr/8-hr light-dark cycle at 18°C. *A. tumefaciens* was freshly streaked out from -80°C glycerol stock onto a 2YT (16 g/L bacto-tryptone, 10 g/L yeast extract, 5 g/L NaCl, pH 7.0) agar plate with proper antibiotics for 2 days at 28°C. A fresh single colony from the plate was used to inoculate 5 ml 2YT liquid medium containing appropriate antibiotics with shaking (220 rpm) at 28°C for 14-18 hr. *Agrobacterium* solution was diluted into 30 ml fresh 2YT medium for growth of 18-24 hr at 28°C until $OD_{600}$ 4.0-5.0. The bacterial suspension was re-suspended in infiltration medium (5% sucrose, 0.01% Silwet, 200 μM AS) at $OD_{600}$ 2. For co-infiltration, the 4-d-old seedlings were covered by *Agrobacterium*-containing infection medium and vacuumed twice for 1 min. Excess infiltration medium was removed and incubated for 3 days at in16-hr/8-hr light-dark cycle at 18°C.

## References


1. Kado CI, Heskett MG: **Selective media for isolation of *Agrobacterium*, *Corynebacterium*, *Erwinia*, *Pseudomonas*, and *Xanthomonas*.** *Phytopathol* 1970, **60:**969-976.
2. Citovsky V, Lee LY, Vyas S, Glick E, Chen MH, Vainstein A, Gafni Y, Gelvin SB, Tzfira T: **Subcellular localization of interacting proteins by bimolecular fluorescence complementation *in planta*.** *J MolBiol* 2006, **362:**1120-1131.
3. Quandt J, Hynes MF: **Versatile suicide vectors which allow direct selection for gene replacement in gram-negative bacteria.** *Gene* 1993, **127:**15-21.
4. Wu HY, Chung PC, Shih HW, Wen SR, Lai EM: **Secretome analysis uncovers an Hcp-family protein secreted via a type VI secretion system in *Agrobacterium tumefaciens*.** *J bacteriol* 2008, **190:**2841-2850.
5. Li JF, Park E, von Arnim AG, Nebenfuhr A: **The FAST technique: a simplified *Agrobacterium*-based transformation method for transient gene expression analysis in seedlings of *Arabidopsis* and other plant species.** *Plant methods* 2009, **5:**6.
6. Marion J, Bach L, Bellec Y, Meyer C, Gissot L, Faure JD: **Systematic analysis of protein subcellular localization and interaction using high-throughput transient transformation of *Arabidopsis* seedlings.** *Plant J* 2008, **56:**169-179.
